# Anticancer Properties of *Phyllanthus emblica* (Indian Gooseberry)

**DOI:** 10.1155/2015/950890

**Published:** 2015-06-09

**Authors:** Tiejun Zhao, Qiang Sun, Maud Marques, Michael Witcher

**Affiliations:** ^1^The Lady Davis Institute The Jewish General Hospital, 3755 Chemin de la Côte-Sainte-Catherine, Montreal, QC, Canada H3T 1E2; ^2^The Departments of Oncology and Experimental Medicine, McGill University, Montreal, QC, Canada H3T 1E2

## Abstract

There is a wealth of information emanating from both *in vitro* and *in vivo* studies indicating fruit extract of the *Phyllanthus emblica* tree, commonly referred to as Indian Gooseberries, has potent anticancer properties. The bioactivity in this extract is thought to be principally mediated by polyphenols, especially tannins and flavonoids. It remains unclear how polyphenols from *Phyllanthus emblica* can incorporate both cancer-preventative and antitumor properties. The antioxidant function of *Phyllanthus emblica* can account for some of the anticancer activity, but clearly other mechanisms are equally important. Herein, we provide a brief overview of the evidence supporting anticancer activity of Indian Gooseberry extracts, suggest possible mechanisms for these actions, and provide future directions that might be taken to translate these findings clinically.

## 1. Introduction

Cancer is a global epidemic with approximately fourteen million new cases being diagnosed each year, leading to an annual death toll of approximately eight million [1]. The societal burden resulting from such staggering numbers is almost immeasurable when one considers both the economic impact and quality of life consequences. However, a recent study puts the economic impact for individual cancers in the billions of dollars for Europe alone [2]. Obviously, more effective therapies are needed for many common cancers. But the holy grail of cancer research would be to identify either pharmacologic or lifestyle interventions that could prevent the onset of cancer. Towards this goal, research has shown that maintaining a healthy lifestyle decreases the risk of some cancers, and intake of some foods may also decrease risk [3–6]. Here, we will focus on the anticancer properties of the fruit-bearing tree* Phyllanthus emblica.*

*Phyllanthus emblica* is a tree indigenous to tropical regions of Southeast Asia. The tree produces a fruit commonly known as Indian Gooseberry or Amla. The* Phyllanthus emblica* fruit (also known as* Emblica officinalis*) or extract from these fruits has been used in traditional medicine for generations to treat symptoms ranging from constipation to the treatment of tumors [7]. Most commonly, the gooseberry was employed as a gentle laxative. However, the potential of* Phyllanthus emblica* extract to be utilized as an anticancer agent has been scrutinized using modern medical techniques over the past two decades. To date, there is substantial evidence that these extracts contain small molecules with both cancer-preventative and antitumor activity. Here, we will provide an overview of the literature supporting these concepts and attempt to lend insight into possible mechanisms whereby the anticancer properties are achieved.

## 2. Cancer Prevention

Each day we are exposed to agents in our environment and through our diet that can potentially compromise the integrity of our genome. Many xenobiotics and the reactive oxygen species generated during cell respiration are carcinogenic. Thus, reducing our exposure to harmful xenobiotics and increasing our capacity to “soak up” reactive oxygen species represented a potential means to reduce the likelihood of cancer. Using the classic measure of carcinogenicity, the Ames test, it was clearly demonstrated that* Emblica officinalis* prevents mutagenesis* in vitro* [8]. Proving this concept* in vivo* is not as straightforward. Measuring cancer prevention in humans is a time consuming and complex endeavor, involving many confounding factors. Fortunately, chemopreventive properties of substances can be tested using rodent models of various cancers.

Taken prophylactically,* Emblica officinalis* extract reduced the genotoxic effects of heavy metals and the carcinogen benzopyrene in murine models [9, 10]. In a murine model of skin carcinogenesis, continuous administration of* Emblica officinalis* extract at 100 mg/kg reduced tumor incidence by ~60% [11]. Similarly, two independent studies showed the polyphenol or aqueous fractions of* Emblica officinalis* administered at 60–250 mg/kg prevented N-nitrosodiethylamine induced hepatocellular carcinoma by ~80–100% [12, 13]. However, such dramatic results were not reported when* Emblica officinalis* extract was examined for chemoprevention of liver tumors induced by initiation with diethylnitrosamine followed by promotion with 2-acetylaminofluorene [14]. This difference indicates* Emblica officinalis* has the capacity to prevent the onset of some, but not all cancers, depending on the initiator. This becomes fairly obvious when one considers carcinogenic compounds have differing modes of action; thus a single extract could not be expected to be universally chemopreventive. Hopefully, future studies will expand on these studies to examine the ability of* Emblica officinalis* to prevent tumors initiated by a wider variety of carcinogens at diverse tissue sites.

How are these extracts chemopreventive? There are four possibilities. First,* Phyllanthus emblica* has potent free radical scavenging activities that might prevent reactive oxygen species induced DNA damage and oncogenesis [15, 16]. However, in the animal models described above, it is unclear to what extent reactive oxygen species contribute to the underlying pathology, implying that there may be alternative mechanisms of action. Second, the extract has properties allowing it to reduce the levels of cytochrome enzymes in liver cells [17]. Cytochromes, such as Cyp 450, convert xenobiotics to potentially carcinogenic substances in an effort to clear them from the body. However, this concept is controversial as Amla extract was not found to decrease Cyp 450 levels in at least two other studies [18, 19]. Third,* Phyllanthus emblica* extracts have anti-inflammatory activities that might prevent inflammation related cancers [20]. Finally, as we will describe below,* Phyllanthus emblica* harbors potent antitumor activity [21, 22]. Even exposure to low levels of extract from these berries may be enough to impair tumor progression at early stages. It should be noted that there is concern regarding potential hepatotoxicity after long term Amla ingestion [23]. This matter may need to be resolved in the future by clinical and epidemiological studies before* Phyllanthus emblica* extract can be safely recommended for long term consumption for the prevention of cancer.

## 3. Cancer Therapy

**Figure 1:**
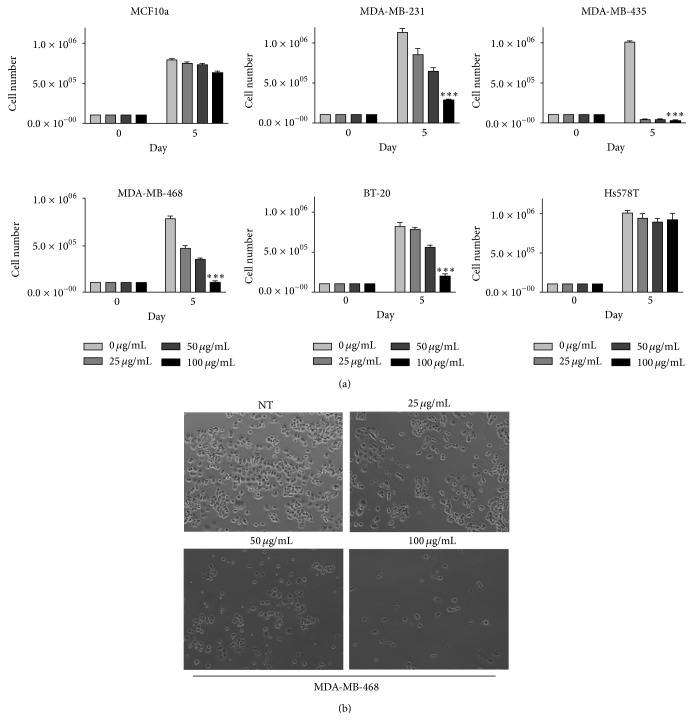
Cytotoxic effects of* Phyllanthus emblica*  (Indian Gooseberry)  extract against triple-negative breast cancer cells. (a) MCF10A cells represent untransformed mammary epithelial cells. All other cell lines represent triple-negative breast cancer cell lines. Growth media for all cell lines were used according to ATCC recommendations. 10^4^ cells were seeded in 24-well plates. 24 hours after plating, Indian Gooseberry extract (Saberry, Sabinsa Corporation) dissolved in PBS was added daily to fresh media at the indicated concentrations or PBS to control cells. Cell viability was measured using trypan blue exclusion with a hemocytometer after a five-day exposure period. Experiments were carried out multiple times in triplicate. Error bars represent SEM.* P* values for growth inhibition of MDA-MB-435, MDA-MB-468, MDA-MB-231, and BT20 cells exposed to 100 *µ*g/mL* Phyllanthus emblica* compared to control cells were all <0.05 (denoted by *∗∗∗*). MCF10A cells exposed to the same concentration showed no significant change. (b) Visualization of MDA-MB-468 cells after five-day exposure to* Phyllanthus emblica* extract at 10x magnification.

*Phyllanthus emblica* extracts have been demonstrated to have potent tumor repressive properties against a number of cancer types both* in vitro* and* in vivo*. Preclinical evidence using a diverse panel of cancer cell lines shows aqueous extract from* Phyllanthus emblica* berries induced apoptosis at concentrations ranging from 50 to 100 micrograms/mL [24]. In this study, normal fibroblasts were also included and showed 4-fold lower sensitivity to these extracts. This is in keeping with our own data comparing aqueous* Phyllanthus emblica* berry extracts (generous gift of Sabinsa Corporation) against triple-negative breast cancer cells (Figures 1(a) and 1(b)). We see exposure of these cells to doses of the soluble extract ranging from 25 to 100 micrograms/mL results in significant cytotoxicity (Figures 1(a) and 1(b)), but almost no effect is seen against normal breast epithelial cells (MCF10A). Other reports have shown extracts from blueberries and strawberries both limit the proliferation of triple-negative breast cancer cells* in vitro* and* in vivo* [25, 26]. However, neither of these extracts showed a considerable degree of antiproliferative activity at concentrations lower than 500 micrograms/mL, whereas soluble* Phyllanthus emblica* berry extract was potent even at 50 micrograms/mL in at least three (MDA-MB-231, MDA-MB-435, and MDA-MB-468) of the cell lines we tested. The sparsity of cells after exposure to the berry extract and appearance of debris indicate cells are undergoing apoptosis as opposed to cytostatic mechanisms of growth arrest. These data indicate* Phyllanthus emblica* extract or a constituent therein represents a potential treatment for breast cancer with low toxicity against nontransformed cells.

*Phyllanthus emblica* berry extract enriched for polyphenols or simple aqueous extracts have also shown cytotoxic activity against cervical and ovarian cancer cells [27, 28]. However, unlike other model systems tested, the reduced proliferation in ovarian cancer cells was attributed to the action of the autophagy pathway, independent of apoptosis [27]. Notably, in contrast to the studies outlined above where* Phyllanthus emblica* prevented liver carcinogenesis,* in vitro* studies using the human liver cancer cell line HepG2 show little evidence of cytotoxicity of aqueous extracts [29]. Overall, it appears that* Phyllanthus emblica* extract displays potent cytotoxic effects against most cell lines, but primary resistance mechanisms exist, as is seen with the Hs578T cell line (Figure 1(a)). Such resistant cells may prove to be a useful tool in determining the mechanism whereby these extracts carry out their cytotoxic effects.

In contrast to the* in vitro* studies, there is sparse evidence regarding the tumor repressive activity of* Phyllanthus emblica* using* in vivo* models of cancer. Such preclinical models are necessary for the translation of these investigations into something clinically relevant. However, a striking study has been carried out employing aqueous extracts of the Indian Gooseberry administered orally against an ovarian cancer xenograft model [27]. In this report, the extracts were able to completely ablate the growth of ovarian xenografts, with little or no residual tumor being observed after treatment. Clearly, these remarkable data warrant further investigation into the* in vivo* anticancer properties of* Emblica officinalis* extracts.

## 4. So, What Is in There?

Extracts from* Phyllanthus emblica* tree have been documented to have a number of disparate properties including antioxidant, anti-inflammatory, proapoptotic, and proautophagy ones [21, 27, 30, 31]. Obviously these berry extracts are a complex mix of compounds [22, 32] and it is not trivial to decipher which molecules mediate these effects. It may be that combinations of molecules from these berries work in synergy with one another to achieve diverse biological activity. Highly purified fractions of low complexity are needed from* Phyllanthus emblica* to be interrogated for each biological property. That being said, some of the known molecules held within the Indian Gooseberryextract, identified by high pressure liquid chromatography (HPLC), can be matched to its anticancer activity [22, 32, 33]. This review will focus on molecules identified by HPLC from* Phyllanthus emblica* extract that have reproducibly been shown to have anticancer properties. Primarily, we will focus on the hydrolyzable tannin content of these extracts because of their high content within the Amla fruit [22] and importantly due to their known antioxidant, cancer-preventative, and cytotoxic activity* in vivo* [34, 35]. However, the flavonoid quercetin perhaps serves as the best paradigm for the future drug development from molecules within Amla extract having anticancer properties (described below), as this molecule has gone to clinical trial and novel analogues are being pursued. While there are other constituents of Amla extract such as vitamin C that have antioxidant activity, the only molecules having bona fide anticancer activity to date fall within the tannin or flavonoid categories.

The major constituents of* Phyllanthus emblica* that have been identified by HPLC and formally characterized as having anticancer activity are described below (and in Table 1). Overall, it seems reasonable that many of the anticancer properties of this fruit are derived from the tannin content. However, a notable exception to this concept is the flavonoid quercetin, which harbors potent antioxidant and anticancer properties as demonstrated utilizing multiple* in vivo* models of cancer including breast and leukemia [36–38].

*Phyllanthus emblica* is rich in polyphenols and hydrolysable tannin derived compounds that act as antioxidants [22, 32]. Well-studied examples include ellagic acid, gallic acid, and chebulagic acid [39–41]. These and other tannins from* Phyllanthus emblica* have been demonstrated to prevent mutagenesis and lipid peroxidation in response to carcinogens and reactive oxygen species [42]. It is likely that the combination of these compounds acting in synergy allows Amla extract to soak up free radicals with such efficiency and imparts the extracts with cancer-preventative properties. Alkylating carcinogens generate DNA mutations through carbon oxidation or conjugation reactions with nucleic acids [43]. It is likely that* Phyllanthus emblica* extract will reduce the oxidative damage induced mutations generated by such agents, but it remains to be seen whether conjugation reactions are also impaired.

There are also similarities between the proapoptotic and autophagy effects of* Phyllanthus emblica* extract and purified components. Most* in vitro* data indicate the antiproliferative effect of these extracts is mediated via activation of an apoptotic cascade. Interestingly, either crude* Emblica officinalis* extracts or purified components are capable of NF-kappaB inhibition [44–47]. Tannins from* Phyllanthus emblica* impairing NF-kappaB inhibition include chebulagic acid, ellagic acid, and corilagin. All of them have been shown to have antiproliferative and proapoptotic properties against cancer cells [48–50]. The same is true for gallic acid, a product of tannin hydrolysis, and major constituent of Amla extract [22]. NF-kappaB is an important survival factor in many cancers, and its inhibition leads to pervasive apoptosis [51, 52]. Thus, many of the anticancer properties of these extracts may be brought about through inhibition of this transcription factor binding with its cognate DNA binding elements. Further, NF-kappaB activation is also a potent inducer of inflammation [53] and again it is reasonable to think that the anti-inflammatory properties of the Amla extract are mediated by NF-kappaB inhibition. However, it should be noted that a least one of the* Phyllanthus emblica* components having potent* in vivo* antitumor activity, pyrogallol, is known to activate reactive oxygen species and NF-kappaB binding [54, 55]. NF-kappaB independent tumor suppression has been described for gallotannin, indicating alternative mechanisms for growth inhibition may be relevant depending on the tissue being targeted [34].

But what about the induction of autophagy? Cell death in response to chebulagic acid and the flavonoid quercetin has been linked to autophagy [27, 56]. Considering apoptosis is tightly linked to the autophagosome, it is likely that many of the tannins present in* Phyllanthus emblica* extract may activate the autophagy and apoptotic response simultaneously [57].

Similar to the tannins outlined in Table 1, quercetin also induces apoptosis and autophagy. However, distinct from tannins, quercetin exhibits a well-characterized inhibition of growth factor signaling pathways. This includes oncogenic signaling pathways such as the EGFR [58, 59]. Quercetin accomplishes this feat through inhibition of PI3K signaling, which plays a pivotal role in relaying oncogenic growth signals from the EGFR. Structural studies of the quercetin analog LY-29004 reveal its activity as a competitive inhibitor of ATP binding to the PI3K catalytic domain [60]. Of course, as is true for any inhibitor, Ly-29004 also has off-target effects, such as CKII inhibition [61], that may play a role in its repression of proliferation.

To become relevant to humans, preclinical data using mouse models of cancer are necessary to interrogate the* in vivo* potential of hydrolyzable tannins. In fact, a number of the components of* Phyllanthus emblica* extract have been demonstrated to have striking anticancer activity against currently incurable cancer using xenograft models. Pyrogallol ablates the growth of lung adenocarcinoma xenografts at only 75 *μ*g/kg [54]. Gallotannin has shown significant tumor response against triple-negative breast cancers and cholangiocarcinoma [34, 62]. Xenograft models of pancreatic and triple-negative breast cancers showed considerable response to ellagic acid [48, 63]. Recent evidence also indicates ellagic acid may act as a prophylactic, protecting against the onset of prostate or breast cancer in animal models [64, 65]. Corilagin demonstrates high antitumor activity against hepatocellular carcinoma xenograft models at 15 mg/kg [66]. Gallic acid shows antitumor qualities against lung and osteosarcoma, but the impact may be not substantial enough to warrant future studies [67, 68].

The flavonoid quercetin has been demonstrated to attenuate tumor growth in multiple animal models. This includes xenograft model of leukemia and pancreatic cancer [38, 69]. Based on the well-defined mechanism of action and encouraging data using murine models, quercetin was tested in a phase I clinical trial where it showed antityrosine kinase activity* in vivo* [70]. Analogues of quercetin designed as more specific PI3K inhibitors also demonstrate potent antiproliferative activities. Such analogues of other small molecules found with the Amla extract may facilitate further development into clinical relevant drugs.

Overall, the data published to date highlights the enormous potential of naturally occurring molecules from* Phyllanthus emblica* as pharmacological agents for the treatment of cancer.

## 5. Future Perspective

For* Phyllanthus emblica* to become relevant clinically, it is imperative that the molecules mediating the antitumor effects of the plant be identified and even more potent, patentable derivatives synthesized. Without the possibility of patents, the pharmaceutical industry will undoubtedly not invest the enormous amount of money required to carry out clinical trials using these putative chemotherapeutics. Such evidenced-based trials will eventually be necessary to prove the worth of these extracts in preventing and treating human cancer.

## Figures and Tables

**Table 1:** Molecules from *Phyllanthus emblica* fruit extract having proven anticancer properties.

Phenolic compounds from *Phyllanthus emblica* extract identified by HPLC having anticancer properties	Cancer model utilized to identify antiproliferative and antitumor properties
Ellagic acid (tannin)	Colon, prostate cell lines, and breast and prostate xenografts

Corilagin (tannin)	Ovarian cancer cells, liver cancer cells, and hepatocarcinoma xenografts

Pyrogallol (tannin)	Lung cancer cells, gastric cancer cells, and lung adenocarcinoma xenografts

Chebulagic acid (tannin)	Retinoblastoma colon cancer, breast cancer, prostate cancer, and leukemia cancer cell lines

Gallic acid (tannin)	Breast and lung cancer cell lines, some activity against lung cancer xenograft

Quercetin (flavonoid)	Numerous cancer cell lines from multiple tissue types, transgenic murine model of breast cancer, leukemia xenograft, and phase I clinical trial
